# A Full Parallel Event Driven Readout Technique for Area Array SPAD FLIM Image Sensors

**DOI:** 10.3390/s16020160

**Published:** 2016-01-27

**Authors:** Kaiming Nie, Xinlei Wang, Jun Qiao, Jiangtao Xu

**Affiliations:** School of Electronic Information Engineering, Tianjin University, 92 Weijin Road, Nankai District, Tianjin 300072, China; nkaiming@tju.edu.cn (K.N.); wangxinlei77@tju.edu.cn (X.W.); qiaojun@tju.edu.cn (J.Q.)

**Keywords:** SPAD, FLIM, CMM, event driven readout, image sensor

## Abstract

This paper presents a full parallel event driven readout method which is implemented in an area array single-photon avalanche diode (SPAD) image sensor for high-speed fluorescence lifetime imaging microscopy (FLIM). The sensor only records and reads out effective time and position information by adopting full parallel event driven readout method, aiming at reducing the amount of data. The image sensor includes four 8 × 8 pixel arrays. In each array, four time-to-digital converters (TDCs) are used to quantize the time of photons’ arrival, and two address record modules are used to record the column and row information. In this work, Monte Carlo simulations were performed in Matlab in terms of the pile-up effect induced by the readout method. The sensor’s resolution is 16 × 16. The time resolution of TDCs is 97.6 ps and the quantization range is 100 ns. The readout frame rate is 10 Mfps, and the maximum imaging frame rate is 100 fps. The chip’s output bandwidth is 720 MHz with an average power of 15 mW. The lifetime resolvability range is 5–20 ns, and the average error of estimated fluorescence lifetimes is below 1% by employing CMM to estimate lifetimes.

## 1. Introduction

Fluorescence lifetime imaging microscopy (FLIM) is a rather new and effective tool that can be used to analyze complex biological samples, either at the microscopic or macroscopic level [1,2]. The progress of confocal microscopy improves the time resolution. The map of fluorescence lifetime allows one to discriminate different fluorophores and to acquire valuable insights into the behavior of emitting molecules, thus obtaining information like local pH and oxygen concentration in cells, *etc.* [3].

The two most common techniques for measuring the fluorescence lifetime are the modulated frequency-domain technique and the time-domain technique [4]. Time-domain techniques include time-correlated single-photon counting (TCSPC) and time-gated technique [5]. TCSPC allows for high accuracy in measuring lifetime and it is the most photon-efficient technique.

In commercial TCSPC systems, one detector, typically an avalanche photodiode (APD) or photomultiplier tube (PMT), with one time-to-digital converter (TDC) measurement channel is raster scanned across a sample. At each point in the image, a laser is pulsed and the arrival time of the first fluorescent photon relative to the laser pulse is measured. With repeated laser pulses, the arrival time of these individual photons is collected and the lifetime is extracted from the exponentially distributed decay curves fitted to the resulting histogram of photon arrival times. Although TCSPC has been developed for many years, several drawbacks still need to be solved. The imaging system is expensive and cumbersome. Compact, high-speed, and portable system-on-chip FLIM solutions which are robust and easy to operate are in increasing demand, especially in clinical and commercial applications [6].

Recently, compact photon counting devices have emerged, known as single-photon avalanche diodes (SPADs) [7,8,9]. As solid-state devices, SPADs can operate in normal atmosphere and at room temperature. The device will not get damaged in detecting strong lights compared with PMT. More importantly, with the development of the SPAD, it can be fabricated in standard CMOS process [10]. The integration of SPADs into standard CMOS process could result in significant system capability of highly parallel single photon detection [11]. Therefore, the SPAD FLIM image sensor is becoming an attractive research field [12]. There are a host of excellent reported works [13,14,15], and most of them are the products of MEGAFRAME project [16,17,18].

Although improved imaging speed has been demonstrated in some of these designs, the parallel acquisition channels lead to very high data throughput that limits the achievable readout frame rates. For photon starved applications such as FLIM, most of the data generated in one frame is useless. So, for higher speed FLIMs, the data compression is necessary. Shepard *et al.* [19] proposed the fastest TCSPC-based fluorescence lifetime imaging system, which is capable of acquiring lifetime images at 100 fps. A data-compression data-path provides a mechanism for efficiently transmitting data off-chip in an event-driven manner.

In addition to the time information, recording precise location is also of importance to the SPAD chip applied in FLIM, and accordingly it is hard to implement event driven readout based on area array image sensors [20]. Henderson *et al.* proposed several high-throughput time-resolved mini-silicon photomultipliers based on the event driven readout technique [21]. Zappa *et al.* proposed a linear 60 × 1 SPAD FLIM array based on the event driven readout [22].

In this work, we propose an area array 16 × 16 SPAD FLIM image sensor, which adopts full parallel event driven readout method by recording the column information, the row information and time information individually. The chip has a fill factor of 5.7%. It can achieve maximum imaging frame rate of 100 fps with a 10 MHz repetition rate excitation laser. The power dissipation of the sensor is 15 mW. The remainder of this paper is organized as follows: Section 2 describes an overview of the image sensor architecture, and the detailed design of each on-chip component. Section 3 shows the analysis of measurement accuracy, highlighting the influence of pile-up effect due to the unique structure. Section 4 shows the circuits simulation results. Section 5 concludes this work.

## 2. TCSPC FLIM Imaging System

### 2.1. Circuits Architecture

**Figure 1:**
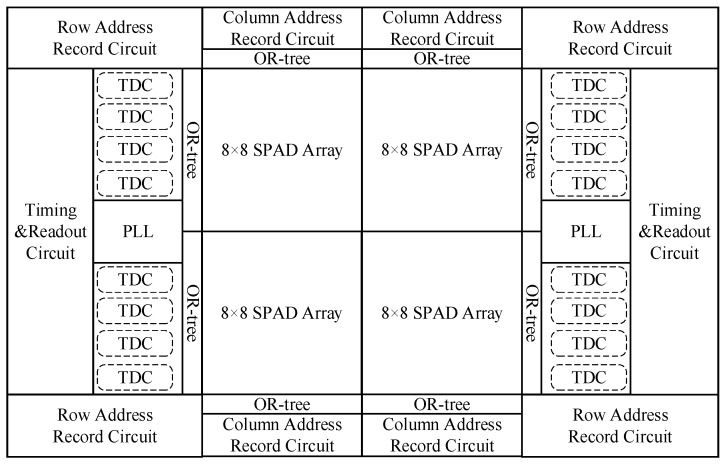
Circuit architecture.

The diagram of the entire system is shown in Figure 1. The resolution is 16 × 16. Internally, each pixel contains a SPAD device, a quenching circuit [23], a calibration circuit and a mono-stable circuit. In each 8 × 8 pixel array, there are corresponding processing circuits, including a TDC array of 4 × 1, a column address recording module and a row address recording module. The entire chip contains two PLLs, in which voltage controlled oscillator (VCO) keeps the same structure and frequency with gated ring oscillator (GRO) in time-to-digital converters (TDCs). The phase-locked loops (PLLs) are in charge of stabilizing the oscillation frequency of GRO, and Process Voltage Temperature (PVT) variations are thus eliminated.

Since the probability of each pixel detecting photons is about 1%, only a few pixels can actually detect photons successfully after one excitation laser pulse. In each excitation period, for the 8 × 8 SPAD array, only four groups of time and position information are recorded. When the chip detects a photon, two OR-trees will lump the time data and the position data from 8 × 8 pixels.

**Figure 2:**
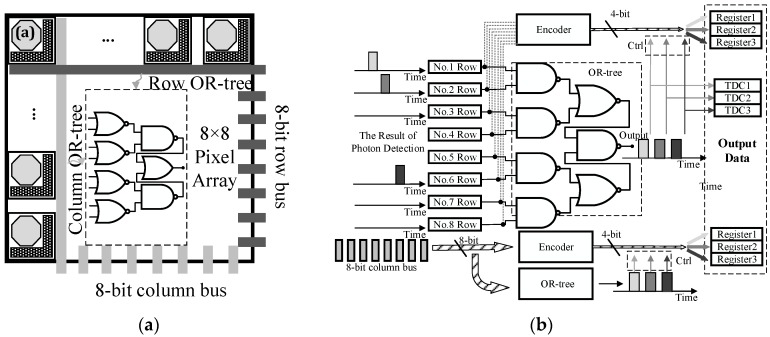
(**a**) The in-pixel OR-tree; (**b**) the operating principle of the event record circuits.

The operating principle of the event record circuits is shown in Figure 2. A group of interleaved token-passing shift registers are used to distribute events to the array of TDCs. The time data of detected photons are transmitted into TDCs in turn, and consequently TDC array can record photons’ arrival time successively [24]. The column and row position data is obtained by two encoders respectively. Then the position data is stored into corresponding registers, which is controlled by the output of the OR-tree. In order to guarantee the validity of data, the data in one frame is valid only when the amounts of column data, row data and time data are the same. By employing event driven readout instead of reading out all data, the data rate is reduced to 1/16 of the initial rate, which significantly mitigate the requirement for input/output (I/O) bandwidth.

### 2.2. SPAD Pixel Array

**Figure 3:**
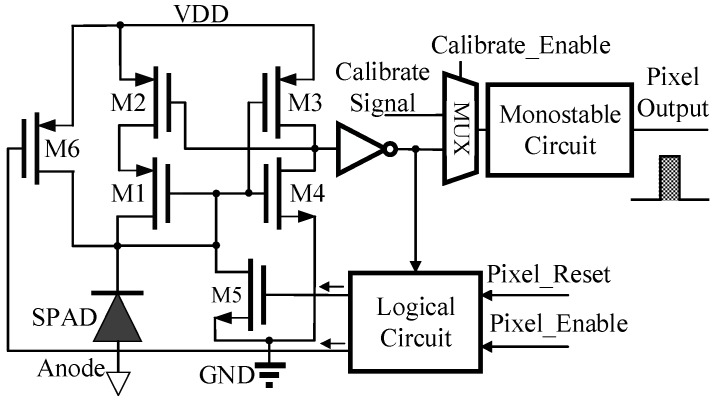
The in-pixel circuits.

In Figure 3, the implementation of the in-pixel circuits is shown. An active quenching circuit based on current sensing is implemented instead of using quenching resistors. After pixels are reset, transistors M1, M2, M3 and M4 form a positive feedback, which can sense the sudden increase of cathode current of the SPAD and quench the current promptly [25]. In addition, the logic circuit controls transistor M5 to speed up the quenching process. After finishing quenching, SPAD does not go back to Geiger mode instantly, but waits for the global RESET signal to open M6. The output voltage of the quenching circuit is converted into a sub-nanosecond voltage pulse through the mono-stable circuit.

Then the pulse is sent through OR-trees of row and column to provide row and column outputs of the 8 × 8 pixel array. The whole 8 × 8 pixel array has 8-bit row bus and 8-bit column bus as outputs. In addition, a calibration module is embedded into pixels in order to calibrate TDC quantization error induced by signal path delay.

### 2.3. Time-to-Digital Converters Array

**Figure 4:**
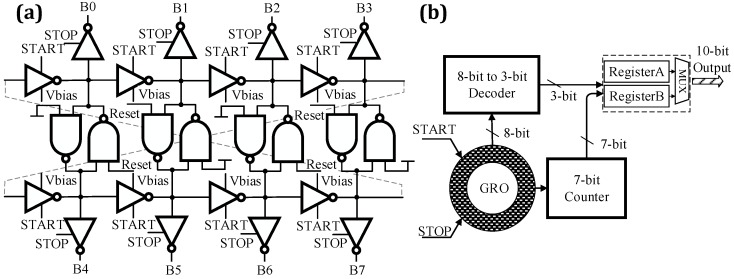
(**a**) The GRO circuit; (**b**) the structure of the 10-bit TDC.

The TDC array, which is similar with the structure shown in [14], has a time resolution of 97.6 ps. The structure of TDC is shown in Figure 4. In order to minimize the TDC’s power consumption, the conversion is achieved in reverse START-STOP mode. The GRO begins to oscillate as soon as the START signal occurs. The global STOP signal is synchronized with the excitation laser. In addition, the oscillator can start oscillating immediately, after the node voltage of the GRO being reset. A 7-bit counter records the number of cycles of the GRO as seven most-significant-bits (MSBs) of the measurement. The three least-significant-bits (LSBs) are provided by the eight states of the GRO. The 10-bit time information is stored into Register A and Register B alternately, and the other register’s data waits to be read out.

**Figure 5:**
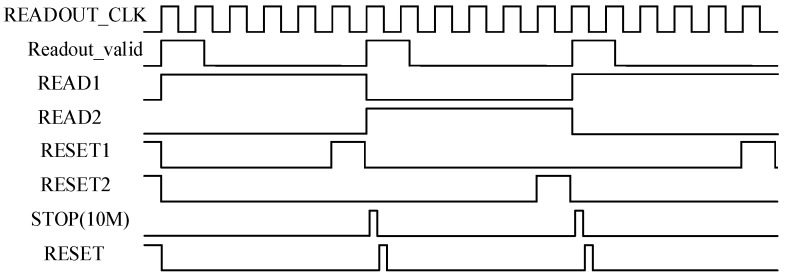
The timing diagram of the scheme.

Figure 5 shows the timing diagram of the scheme. The RESET signal is used to reset TDCs and pixels. The READ1, READ2, RESET1 and RESET2 signals are used to control the two groups of registers in each TDC alternately.

## 3. Fluorescence Lifetime Imaging Error Analysis for the Proposed Readout Method

### 3.1. Pile-Up Effect during Events Readout

**Figure 6:**
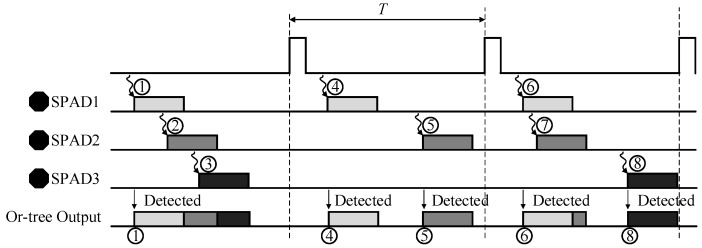
The schematic diagram of pile-up effect.

The readout circuits of this chip lead to the result that not every pulse can be detected by TDC arrays or position recording circuits. The pile-up effect of TCSPC is classified into three types. The first one is the traditional pile-up effect and it can be alleviated by reducing photon-rate to 1%, and the photon-rate is directly proportional to the emission intensity. The second one is caused by the dead time of SPAD devices. Nonetheless, thanks to the global reset of the active quenching circuits, this part can be neglected. The last one is the dead time of the processing circuits of the chip, *i.e.*, the time interval between the photon’s arrival and being detected. The chip proposed in this paper is influenced mostly by the third kind of pile-up effect. Pulses from each pixel are shortened by the mono-stable circuit but still have a finite length *t*_p_. For the 8 × 8 pixel array, if the time interval of any two photon events is less than *t*_p_ apart, two pulses will merge together at the output of the OR-tree. Consequently, only the first event will get processed further and the second event is missed completely. Figure 6 shows the process of an 8 × 8 pixel array detecting photons. It can be seen that the electrons labelled as 2, 3 and 7 will not be detected due to the dead time of the OR-tree. Thus, the photons’ arrival time histogram is modulated by the readout circuits.

Another influencing factor to be considered in the detecting behavior is the interactions among pixels within the same sub-array and it is different from the common cross-talk phenomenon in device level. It derives from the variation of the connecting probability towards TDCs. The probability is modulated by the neighboring pixels. If the sensor is supposed to work as a Mini-Silicon Photomultiplier (MSP), the lifetimes that each pixel needs to measure are the same; therefore, the interactions among the sub-array are also the same. Hence, the influence of interactions can be neglected. However, if the sensor is supposed to work as an area array detector, each pixel may need to detect different lifetimes. Short lifetime means a rapid decay and most photons are expected to be detected in a short period after excitation. Long lifetime is in the opposite condition. This results in a larger probability that the TDC channels are occupied by pixels that detect short lifetimes. The pile-up effect is thus deteriorated. Furthermore, the modulated influence along the whole detecting period is not the same. In order to simplify the analysis, the fluorescence is assumed to have a single-exponential decay. The influence is analyzed and simulated in Maximum Likelihood Estimator (MLE), Center-of-Mass Method (CMM) and Least-Squares Method (LSM). When the sensor works as a MSP, the average number of photons that one pixel can detect is [26]:

(1) $$c_{aver}(\mu ;t)=\frac{1}{N^{2}\tau }\exp(-t/\tau )\times \left\{ \begin{array}{ll} \exp(-\mu (1-e^{-t/\tau })) & t<t_{p}\\ \exp(-\mu e^{-t/\tau }(e^{t_{p}/\tau }-1)) & \;t\geq t_{p} \end{array}\right.$$

where *τ* is the lifetime, *N*^2^ is the number of pixels and *μ* is the expected number of photons the pixel array detects.

When the sensor works as an area array detector, we only analyze one 8 × 8 pixel array because each 8 × 8 pixel array is independent. Assuming that *τ*_i,j_ is the lifetime which pixel (*i*, *j*) needs to detect, and then the probability of photon detection on pixel (*i*, *j*) is:

(2) $$P_{i,j}(t)=\frac{P0_{i,j}}{\tau_{i,j}}\exp(-t/\tau_{i,j})$$

where *P*0_i,j_ is the photon-rate of pixel (*i*, *j*). Then the probability failing to detect photon on pixel (*i*, *j*) is:

(3) $$P_{i,j}^{'}(t;t_{p})=\left\{ \begin{array}{ll} 1-P0_{i,j}(1-\exp(-t_{p}/\tau_{i,j})) & t<t_{p}\\ 1-P0_{i,j}(\exp(-(t-t_{p})/\tau_{i,j})-\exp(t/\tau_{i,j})) & t\geq t_{p} \end{array}\right.$$

If the interaction of other pixels is neglected, then for a certain pixel, which is assumed as pixel (1, 1), the probability of photon detection along with time is:

(4) $$P_{1,1}^{'}(t,t_{p})=\prod_{i=2}^{n}\prod_{j=2}^{n}P_{i,j}^{'}(t,t_{p})P_{1,1}(t)$$

To simulate the influence of pile-up effect, the Monte Carlo simulation of the operation process of the chip is done in MATLAB. The MATLAB random number generator is used to simulate individual photon events with the appropriate statistical distribution. For a single-exponential decay, the probability that the pile-up occurs can be analyzed.

**Figure 7:**
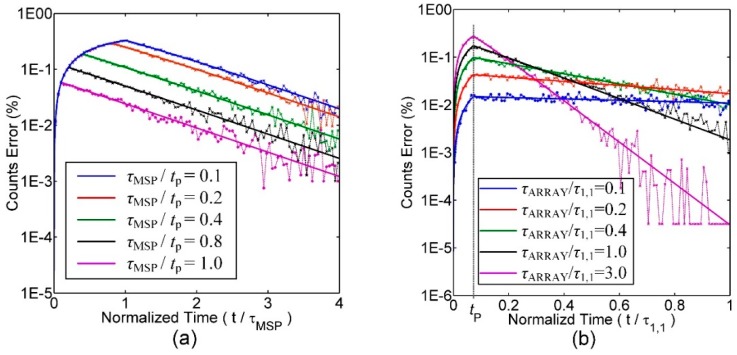
(**a**) The counts errors of events along normalized time under different *τ*_MSP_/*t*_p_; (**b**) the counts errors of events along normalized time under different *τ*_ARRAY_/*τ*_1,1_.

Figure 7a,b is the simulation results of the counts error of events in different measuring windows on conditions that the pixel working under different *τ*_MSP_/*t*_p_ and *τ*_ARRAY_/*τ*_1,1_, respectively, where *τ*_MSP_ is the lifetime being measured by the pixel array when it is used as a MSP, *τ*_1,1_ is the lifetime being measured by the pixel (1, 1) when the SPAD array is used as an array sensor, and *τ*_ARRAY_ is the lifetime being measured by other pixels. The dashed line is the simulated data, while the solid line is the theoretical curve. From the two figures, it can be seen that the simulation results keep in accordance with the theory anticipation. During the period close to the excitation, it is more probable for the pixel array to detect photons, so the pile-up effect is more apparent and the counts error increases. After a while, the probability of photon detection falls, so the distribution gradually approaches to single-exponential curve. In PMT working mode, as *τ*_MSP_/*t*_p_ goes smaller, the pile-up effect gets degraded. But when the sensor works as an array imager, the influence is modulated along time. When *τ*_ARRAY_/*τ*_1,1_ = 0.1, pixel (1,1) detects longer lifetimes than other pixels do. Then at the beginning of the detection, pile-up effect from pixel (1,1) is less and the counts error is comparatively small. However, in later detection time, pile-up effect from pixel (1,1) becomes strong and the counts error is comparatively large. In the simulation process of the chip circuits, *t*_p_ is found to be 360 ps.

### 3.2. Fluorescence Lifetime Imaging Algorithm

In this section, the influence of pile-up effect to MLE, CMM and LSM is simulated and analyzed. For a fluorescence histogram with a single-exponential decay *f*(*t*) = *A*exp(−*t*/*τ*) in a measurement window 0 ≤ *t* ≤ *T* recorded by the 10-bit TDC (*M* = 1024), the lifetime estimated using MLE, *τ*_MLE_, can be obtained by [27]:

(5) $$1+{\left\{\exp(h/\tau_{MLE})-1\right\}}^{-1}-M{\left(\exp(Mh/\tau_{MLE})-1\right)}^{-1}=\sum_{j=1}^{M}jN_{j}/N_{c}$$

where *N_c_* is the total signal counts within the measurement window, *h* is the LSB of TDC, *N_j_* is the number of recorded counts in the *j*th time bin (*j* = 1, 2, ... , *M*).

The CMM can be viewed as a hardware implementation algorithm of the MLE although their physical definitions are not the same. The CMM is easy to be implemented by FPGA [28], and then it is possible to achieve video-rate fluorescence lifetime imaging by employing CMM to estimate fluorescence lifetimes. The lifetime estimated using CMM, *τ*_CMM_, can be obtained by:

(6) $$\tau_{\text{CMM}}=\left(\frac{\sum_{j=1}^{M}jN_{j}}{N_{c}}+\frac{1}{2}\right)h$$

The LSM minimizes the chi-square, ∑{(*o* − *e*)^2^/*e*}, where *o* is the statistics value and *e* is the expected value. The lifetime estimated using LSM, *τ*_LSM_, can be obtained by:

(7) $$\frac{\exp(T/\tau_{LSM})}{\exp(T/\tau_{LSM})-1}-\frac{M}{\exp(MT/\tau_{LSM})-1}=\frac{\sum_{j=1}^{M}jN_{i}^{2}\exp(jT/\tau_{LSM})}{\sum_{j=1}^{M}N_{i}^{2}\exp(jT/\tau_{LSM})}$$

**Figure 8:**
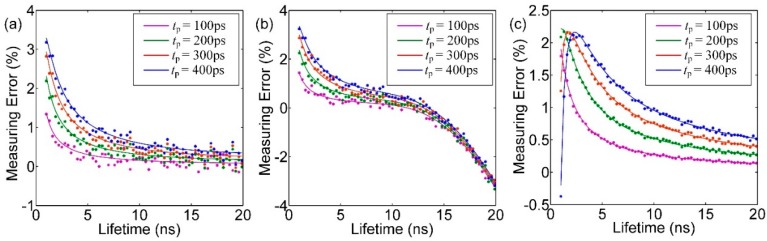
The relationship between measuring error of different lifetimes and *t*_p_ while using (**a**) MLE; (**b**) CMM; (**c**) LSM to estimate lifetimes.

Figure 8 illustrates the impact of pile-up effect on estimating lifetime using MLE, CMM and LSM respectively, when the pixel array measures uniform lifetime. The theoretical results marked as solid lines are compared to Monte Carlo simulations marked with asterisks (scattered points). It can be seen that there is not much difference between MLE and CMM under condition that the lifetime being measured is short. But when the lifetime being measured gets longer, it cannot be guaranteed that all photons triggered by the laser can be detected by the sensor due to the limited detection time window. As a result, the measuring error of CMM increases. From Figure 8, it also can be seen that *t*_p_ is the dominant factor that influences the measurement accuracy.

**Figure 9:**
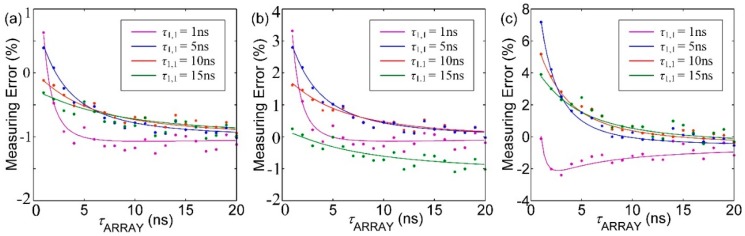
The relationship between measuring errors and *τ*_ARRAY_ while using (**a**) MLE; (**b**) CMM; (**c**) LSM to estimate lifetimes.

Figure 9 shows the interaction effect of pixels under condition that the chip works as an area array image sensor. In this figure, it is assumed that *τ*_1,1_ is 1 ns, 5 ns, 10 ns and 15 ns respectively. Also, the measuring error is relative to *τ*_1,1_. When *τ*_ARRAY_ is short, the pile-up effect becomes degraded and the deviation is serious. Considering the situation that *τ*_1,1_ is 5 ns and *τ*_ARRAY_ is 1 ns, the deviation reaches 9% by employing LSM. To maintain the average estimated error below 1% in CMM algorithm, the detecting range of the pixels are 5–20 ns. MLE maintains its accuracy below 1% in full range. But MLE requires massive calculations and is not practical through hardware.

The hold time of encoders also influences the accuracy of measurements. When the time interval of two events is shorter than the hold time of encoders, the encoders used to record the column and row data of events cannot get the correct position code of the first event. Once that occurs, the output code of encoder is “1000”, the data of this time of fluorescence trigger is abandoned.

The resolvability range of this TCSPC design depends on the error from the estimation algorithm and the influencing factors from detecting operation. CMM typically has an ideal resolvability range of *T*/4 to *T*/100 with post software calibrations where *T* is the period of the laser pulse. The origination of this algorithm error is Poisson noise [28]. The influencing factors, such as the pulse width *t*_p_ output from mono-stable circuit and the interaction effect among pixels, also shorten the detecting range. In this design, after an overall consideration, the resolvability range is approximately 5–20 ns under 10 MHz laser excitation rate.

The detecting range in this design is suitable in applications of long decay lifetimes. However, in actual biomedical applications such as Indocyanine Green (ICG), the resolvability range of sub-nanosecond is of great importance. The design should be optimized to satisfy the extending range. First, the quantization range of TDC should be enough as the reverse arrival time of photons becomes longer under rapid decays. Then, the module delays also need to be minimized to accelerate the response. Additionally, the number of TDCs shared among the same sub-array should be enlarged to alleviate pile-up effects.

**Figure 10:**
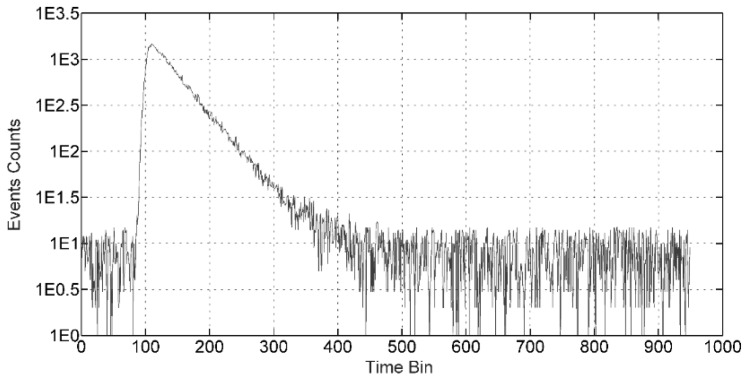
The events distribution under condition that background and DCR are considered.

In actual FLIM measurements, the existence of background and DCR will restrict the accuracy of measurement. As background and DCR are rather lower than the signal intensity, as seen in Figure 10, so the influence to the pile up effect can be ignored. Background and DCR are taken into account by the method mentioned in [28].

## 4. Simulation Results of Circuits

The circuit design is based on the 0.13 μm 1P3M CIS process and SPADs are substituted by a Verilog A model. The Verilog A model has the advantage of directly generating random events obeying the exponential distribution. Synchronized to 10 MHz laser excitations, the proposed structure of a 16 × 16 array is simulated to detect various lifetimes with 1% detecting probability. The resolution of two column-level TDCs is 97 ps. We should take note that the resolution of the SPAD array is not constrained below 16 × 16. The SPAD array can be extended by placing more 8 × 8 sub-arrays with the same TDC/SPAD ratio. However, too large array may be problematic in layout routing. The design of 16 × 16 SPAD array in this work is to testify the proposed readout method in fast imaging mode.

**Figure 11:**
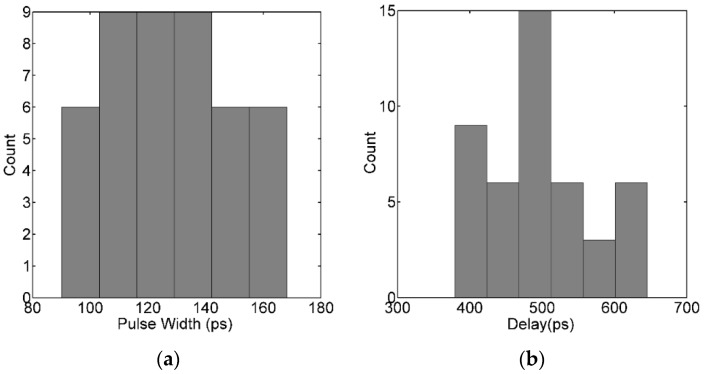
The process corner simulation result of the quenching circuit: (**a**) *t*_width_; (**b**) *t*_delay_.

The pixel is influenced by process, voltage and temperature variations. Figure 11 is the process corner simulation result of the quenching circuit, where *t*_delay_ is the time delay between the output pulse of pixels and the photon’s arrival time and *t*_width_ is the width of the output pulse. The process corner covers ‘ss (NMOS: Slow and PMOS: Slow)’ to ‘ff (NMOS: Fast and PMOS: Fast)’ and the temperature is swept from −40 °C to 80 °C. The simulation results show that *t*_width_ varied from 90 ps to 170 ps, and the *t*_delay_ varied from 400 ps to 650 ps. The deviation of the process corner contributes to the estimation error of lifetimes.

**Figure 12:**
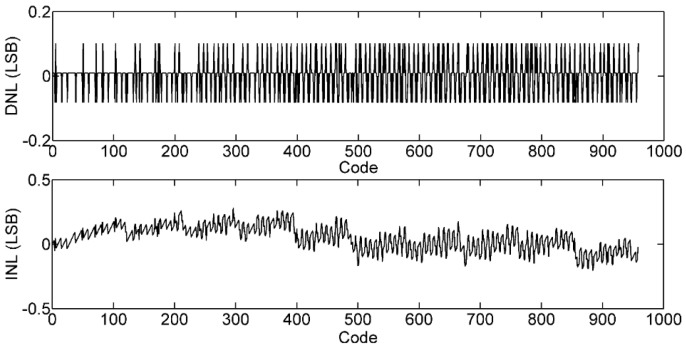
The DNL and INL of the proposed TDC.

The non-linearity of the proposed TDC is analyzed with an input of ramp signal. Figure 12 is the linearity result of the proposed TDC. As the actual time range that the TDC needs to quantify is 0–95 ns, the digital output of the proposed TDC is limited to 950. The differential nonlinearity (DNL) is −0.082LSB\0.102LSB, and the integral nonlinearity (INL) is −0.205\0.282 LSB.

During the FLIM simulation, it is assumed that the fluorescence intensity of the whole picture is uniform, which means that the probability of every pixel to detect photons is 1%. This leads to the fact that the simulation result is worse than the actual measured result because the impact of pile-up is maximized. The maximum imaging frame rate is 100 fps, in the situation that a lifetime map can be obtained by handling the information of about 1000 photons each pixel. If the accurate imaging is needed, the imaging frame rate can be decreased to reduce the standard deviation of estimated lifetimes.

**Figure 13:**
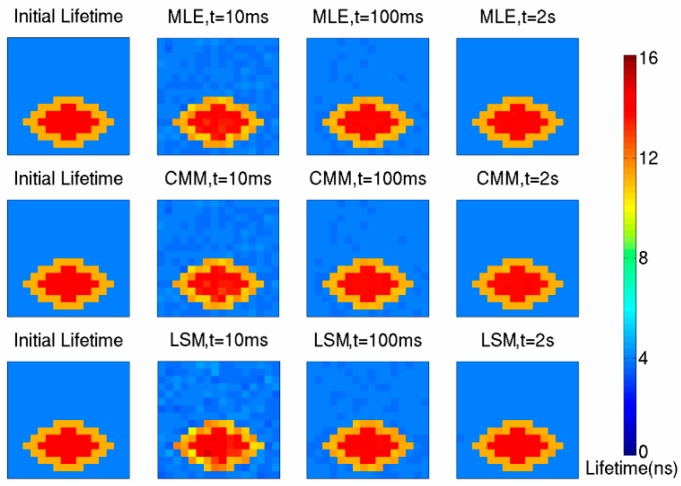
The simulation result of No. 1 picture.

The No. 1 picture (the initial lifetime map in Figure 13) used as fluorescence source has a fluorophore whose lifetime is 14 ns, and the background lifetime is 4 ns. The total exposure time is 10 ms, 100 ms and 2 s, respectively, and the output data of the circuits is handled by MLE, CMM and LSM, respectively. The simulation results are shown in Figure 13. It can be seen that the outline of the fluorophore is obvious when the total exposure time is 10 ms, which means that the imaging frame rate can be 100 fps.

**Figure 14:**
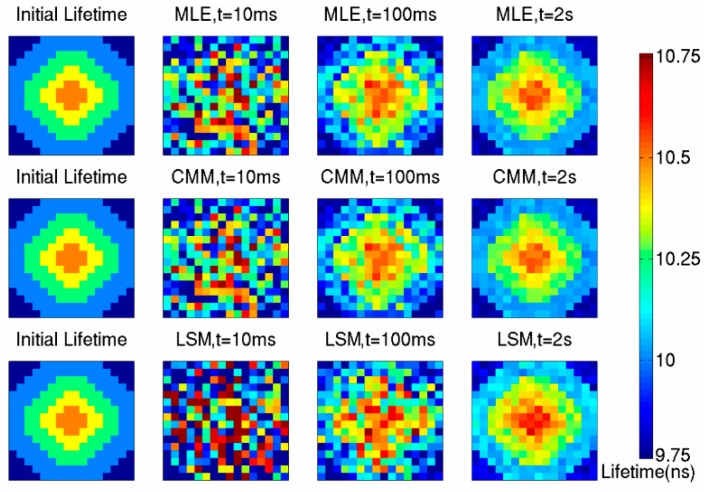
The simulation result of No. 2 picture.

The No. 2 picture (the initial lifetime map in Figure 14) used as fluorescence source has a fluorophore whose lifetime is 10.5 ns. The simulation results are shown in Figure 14. The outline of fluorophores becomes difficult to distinguish when the imaging frame rate approaches 100 fps, the variance of fluorescence lifetime imaging results no longer stands negligible. In the circumstance of 10 fps, namely 100 ms exposure time, the fluorophore’s profile can be distinguished.

Table 1 shows the mean values, variances and FoMs of detecting results of pixel arrays with 10.5 ns lifetime. The Figure of Merit (FoM) of fluorescence lifetime imaging can be expressed as follows:

(8) $$FoM=\frac{\tau }{\sqrt{{\sigma_{\tau }}^{2}+\Delta \tau^{2}}}$$

where *σ_τ_* is the standard deviation of estimated lifetimes, and Δ*τ* is the average offset of estimated lifetimes.

**Table 1:** The simulation results of lifetimes of 10.5 ns.

	MLE	CMM	LSM
Actual Lifetime [ns]	10.5	10.5	10.5
Estimated Lifetime [ns]	10.537	10.53	10.589
Average Error [%]	0.35	0.28	0.84
Standard Deviation@(10 ms)[ps]	413	410	672
Standard Deviation@(100 ms)[ps]	34.9	34.6	115
Standard Deviation@(2 s)[ps]	23	22.9	46
Imaging FoM@(2 s)	241	279	105

Table 2 summarizes the detailed simulation results of the estimated fluorophore’s lifetime. The volatility of FoM is attributed to the lack of sample volume. Since the simulation of circuits takes a lot of time, only 20 M cycles are simulated. It can be concluded that the FoM of LSM is rather smaller than MLE and CMM. As the fluorescence lifetime falls in between 5 ns and 14 ns, CMM is close to MLE in terms of FoM, but when it comes to 14 ns or longer, CMM’s FoM presents apparent decrease.

**Table 2:** The simulation results of lifetimes of 14 ns.

	MLE	CMM	LSM
Actual Lifetime [ns]	14	14	14
Estimated Lifetime [ns]	14.03	13.95	14.24
Average Error [%]	0.24	0.28	1.69
Standard Deviation@(10 ms) [ps]	442	423	487
Standard Deviation@(100 ms) [ps]	119	114	158
Standard Deviation@(2 s) [ps]	36.9	35.3	45
Imaging FoM@(2 s)	280	242	59

The simulation results above indicate that moderate measurement accuracy is attainable using full parallel event driven readout method. The CMM and MLE algorithm can be put in use for estimating fluorescence lifetimes. MLE is capable of extending the lifetime resolvability range with a high performance computer. But the CMM shows its advantage in the easy implementation of hardware.

Table 3 summarizes the performance of the chip and shows the comparisons with other works. A slow repetition rate excitation laser is used in order to extend the lifetime resolvability range. The low power dissipation is attributed to the reduced number of TDCs. The chip’s maximum readout sample rate is 10 MSps, which is faster than that reported in all except one of the previously published articles that are referenced in Table 3. The frame rate is defined as the lifetime generation rate. The lifetime is estimated from the histograms consisting of large number of photons. The maximum frame rate in this work is 100 fps. The less number of collected photons means a lower signal-to-noise ratio compared with previous work in Reference [14] or [18]. Meanwhile, the output bandwidth is reduced to 720 Mbps. The Figure of Merit (FoM) of the proposed chip can be expressed as follows:

(9) $$FoM=\frac{Power}{Resolution\cdot Readout\_Sample\_Rate}$$

The FoM of the proposed structure is 5.86 pJ/(sample·pixel). The extremely low chip FoM is attributed to the implementation of full parallel event-driven readout, the small resolution and the reduction of measuring accuracy.

**Table 3:** Comparisons of different architectures.

Parameters	Reference 14	Reference 18	Reference 15	Reference 19	This Work
Resolution	160 × 128	32 × 32	64 × 64	64 × 64	16 × 16
CMOS Technology	0.13 um	0.13 um	0.35 um	0.13 um	0.13 um
Fill Factor [%]	1	2	0.93	0.77	5.7
Number of TDC	1/1	1/1	4096/1	1/1	64/4
LSB of TDC [ps]	55	119	350	62.5	97
Laser Pulse Rate [MHz]	40	40	N/A	20	10
Sampling Rate [Sps]	50 k	1 M	718	20 M	10 M
Lifetime frame rate [fps]	0.08	1.8	3.9	100	100
@ histogram photons	@ ≈ 30,0000	@ ≈ 22,0000	@ ≈ 200	@ ≈ 600	@ = 1000
Bandwidth [bps]	51.2 G	10.24 G	N/A	42 G	720 M
Power [W]	550 m	90 m*	1.4	8.79	15 m**
Chip FoM [pJ/(sample·pixel)]	537	87	476 k	107	5.86

* Without I/O pads. ** Simulation result of core power consumption.

## 5. Conclusions

In this work, we have proposed a full parallel event driven readout method which is implemented in the area array SPAD image sensor for high-speed FLIM. The maximum imaging frame rate is 100 fps, and the number of TDCs used declined to 16. The average power consumption is 15 mW. The output bandwidth is reduced to 720 Mbps. We did the analysis of imaging error caused by pile-up effect which is induced by the readout method. The lifetime resolvability range is 5–20 ns, and the average error of estimated fluorescence lifetimes is below 1%. The proposed readout method is suitable in video-rate FLIM with the resolvability of long decay lifetimes.

## References

[1] Siegel J., Elson D.S., Webb S.E.D., Lee K.C.B., Vlandas A., Gambaruto G.L., Lévêque-Fort S., Lever M.J., Tadrous P.J., Stamp G.W.H. (2003). Studying biological tissue with fluorescence lifetime imaging: Microscopy, endoscopy, and complex decay profiles. Appl. Opt..

[2] Fatakdawala H., Poti S., Zhou F., Sun Y., Bec J., Liu J., Yankelevich D.R., Tinling S.P., Gandour-Edwards R.F., Farwell D.G. (2013). Multimodal *in vivo* imaging of oral cancer using fluorescence lifetime, photoacoustic and ultrasound techniques. Biomed. Opt. Express..

[3] Cubeddu R., Comelli D., D’Andrea C., Taroni P., Valentini G. (2002). Time-resolved fluorescence imaging in biology and medicine. J. Phys. D Appl. Phys..

[4] Pawley J.B. (1995). Handbook of Biological Confocal Microscopy.

[5] Mosconi D., Stoppa D., Pancheri L., Gonzo L., Simoni A. CMOS Single Photon Avalanche Diode Array for Time-Resolved Fluorescence Detection. Proceedings of the 32nd European Solid-State Circuits Conference (ESSCIRC).

[6] Li D., Arlt J., Richardson J., Walker R., Buts A., Stoppa D., Charbon E., Henderson R. (2010). Real-time fluorescence lifetime imaging system with a 32 × 32 0.13 μm CMOS low dark-count single-photon avalanche diode array. Opt. Express..

[7] Savuskan V., Gal L., Cristea D., Javitt M., Feiningstein A., Leitner T., Nemirovsky Y. (2015). Single photon avalanche diode collection efficiency enhancement via peripheral well controlled field. IEEE Trans. Electron Dev..

[8] Cova S., Ghioni M., Lacaita A., Samori C., Zappa F. (1996). Avalanche photodiodes and quenching circuits for single-photon detection. Appl. Opt..

[9] Richardson J.A., Webster E.A.G., Grant L.A., Henderson R.K. (2011). Scaleable Single-Photon Avalanche Diode Structures in Nanometer CMOS Technology. IEEE Trans. Electron Dev..

[10] Palubiak D.P., Deen M.J. (2014). CMOS SPADs: Design Issues and Research Challenges for Detectors, Circuits, and Arrays. IEEE J. Sel. Top. Quantum Electron..

[11] Stoppa D., Mosconi D., Pancheri L., Gonzo L. (2009). Single-Photon Avalanche Diode CMOS Sensor for Time-Resolved Fluorescence Measurements. IEEE Sens. J..

[12] Li D.D., Ameerbeg S.M., Arlt J., Tyndall D., Walker R.J., Matthews D.R. (2012). Time-Domain Fluorescence Lifetime Imaging Techniques Suitable for Solid-State Imaging Sensor Arrays. Sensors.

[13] Vitali M., Bronzi D., Krmpot A.J., Nikolić S.N., Schmitt F.J., Junghans C., Tisa S., Friedrich T., Vukojević V., Terenius L. (2014). A Single-Photon Avalanche Camera for Fluorescence Lifetime Imaging Microscopy and Correlation Spectroscopy. IEEE J. Sel. Top. Quantum Electron..

[14] Veerappan C., Richardson J., Walker R., Li D.D.U., Fishburn M.W., Maruyama Y., Stoppa D., Borghetti F., Gersbach M., Henderson R.K. A 160 × 128 single-photon image sensor with on-pixel 55 ps 10 b time-to-digital converter. Proceedings of the IEEE International Solid-state Circuits Conference (ISSCC).

[15] Schwartz D.E., Charbon E., Shepard K.L. (2008). A Single-Photon Avalanche Diode Array for Fluorescence Lifetime Imaging Microscopy. IEEE J. Solid State Circuits.

[16] Stoppa D., Borghetti F., Richardson J., Walker R., Grant L., Henderson R.K., Gersbach M., Charbon E. A 32 × 32-pixel array with in-pixel photon counting and arrival time measurement in the analog domain. Proceedings of the European Solid-state Circuits Conference (ESSCIRC).

[17] Niclass C., Favi C., Kluter T., Gersbach M., Charbon E. (2008). A 128 × 128 single-photon image sensor with column-level 10-bit timeto-digital converter array. IEEE J. Solid State Circuits.

[18] Gersbach M., Maruyama Y., Trimananda R., Fishburn M.W., Stoppa D., Richardson J.A., Walker R., Henderson R., Charbon E. (2012). A Time-Resolved, Low-Noise Single-Photon Image Sensor Fabricated in Deep-Submicron CMOS Technology. IEEE J. Solid State Circuits.

[19] Field R.M., Realov S., Shepard K.L. (2014). A 100 fps, Time-Correlated Single-Photon-Counting-Based Fluorescence-Lifetime Imager in 130 nm CMOS. IEEE J. Solid State Circuits.

[20] Homulle H. (2014). Development of a Multichannel TCSPC System in a Spartan 6 FPGA. Ph.D. Thesis.

[21] Dutton N.A.W., Gnecchi S., Parmrsan L., Holmes A.J., Rae B., Grant L.A., Henderson R.K. A Time-Correlated Single-Photon Counting Sensor with 14 GS/s Histogramming Time-to-Digital Converter. Proceedings of the IEEE International Solid-state Circuits Conference (ISSCC).

[22] Villa F., Lussana R., Tosi A., Zappa F. (2015). High fill-factor 60 × 1 SPAD array with 60 sub-nanosecond integrated TDCs. IEEE Photonic Tech. Lett..

[23] Stipčević M. (2009). Active quenching circuit for single-photon detection with Geiger mode avalanche photodiodes. Appl. Opt..

[24] Tyndall D., Rae B.R., Li D.D.U., Arlt J., Johnston A., Richardson J.A., Henderson R.K. (2012). A High-Throughput Time-Resolved Mini-Silicon Photomultiplier With Embedded Fluorescence Lifetime Estimation in 0.13 μm CMOS. IEEE Trans. Biomed. Circuits Syst..

[25] Mita R., Palumbo G. (2008). High-Speed and Compact Quenching Circuit for Single-Photon Avalanche Diodes. IEEE Trans. Instrum. Meas..

[26] Arlt J., Tyndall D., Rae B.R., Li D.D.U., Richardson J.A., Henderson R.K. (2013). A study of pile-up in integrated time-correlated single photon counting system. Rev. Sci. Instrum..

[27] Hall P., Sellger B. (1981). Better Estimates of Exponential Decay Parameters. J. Phys. Chem..

[28] Li D.D.U., Arlt J., Tyndall D., Walker R., Richardson J., Stoppa D., Charbon E., Henderson R.K. (2011). Video-rate fluorescence lifetime imaging camera with CMOS single-photon avalanche diode arrays and high-speed imaging algorithm. J. Biomed. Opt..

